# Paraoxonase-1 Regulation of Renal Inflammation and Fibrosis in Chronic Kidney Disease

**DOI:** 10.3390/antiox11050900

**Published:** 2022-04-30

**Authors:** Fatimah K. Khalaf, Chrysan J. Mohammed, Prabhatchandra Dube, Jacob A. Connolly, Apurva Lad, Usman M. Ashraf, Joshua D. Breidenbach, Robin C. Su, Andrew L. Kleinhenz, Deepak Malhotra, Amira F. Gohara, Steven T. Haller, David J. Kennedy

**Affiliations:** 1 Department of Medicine, College of Medicine and Life Sciences, University of Toledo, Toledo, OH 43606, USA; kareem.khalaf@utoledo.edu (F.K.K.); chrysan.mohammed@rockets.utoledo.edu (C.J.M.); prabhatchandra.dube@utoledo.edu (P.D.); jacob.connolly@rockets.utoledo.edu (J.A.C.); apurva.lad@utoledo.edu (A.L.); usman.ashraf2@rockets.utoledo.edu (U.M.A.); joshua.breidenbach@rockets.utoledo.edu (J.D.B.); robin.su@rockets.utoledo.edu (R.C.S.); andrew.kleinhenz@utoledo.edu (A.L.K.); deepak.malhotra@utoledo.edu (D.M.); amira.gohara@utoledo.edu (A.F.G.); steven.haller@utoledo.edu (S.T.H.); 2 Department of Clinical Pharmacy, University of Alkafeel, Najaf 54001, Iraq

**Keywords:** paraoxonase-1, fibrosis, inflammation, chronic kidney disease

## Abstract

Papraoxonase-1 (PON1) is a hydrolytic lactonase enzyme that is synthesized in the liver and circulates attached to high-density lipoproteins (HDL). Clinical studies have demonstrated an association between diminished PON-1 and the progression of chronic kidney disease (CKD). However, whether decreased PON-1 is mechanistically linked to renal injury is unknown. We tested the hypothesis that the absence of PON-1 is mechanistically linked to the progression of renal inflammation and injury in CKD. Experiments were performed on control Dahl salt-sensitive rats (SS^Mcwi^, hereafter designated SS rats) and Pon1 knock-out rats (designated SS-Pon1^em1Mcwi^, hereafter designated SS-PON-1 KO rats) generated by injecting a CRISPR targeting the sequence into SS^Mcwi^ rat embryos. The resulting mutation is a 7 bp frameshift insertion in exon 4 of the PON-1 gene. First, to examine the renal protective role of PON-1 in settings of CKD, ten-week-old, age-matched male rats were maintained on a high-salt diet (8% NaCl) for up to 5 weeks to initiate the salt-sensitive hypertensive renal disease characteristic of this model. We found that SS-PON-1 KO rats demonstrated several hallmarks of increased renal injury vs. SS rats including increased renal fibrosis, sclerosis, and tubular injury. SS-PON-1 KO also demonstrated increased recruitment of immune cells in the renal interstitium, as well as increased expression of inflammatory genes compared to SS rats (all *p* < 0.05). SS-PON-1 KO rats also showed a significant (*p* < 0.05) decline in renal function and increased renal oxidative stress compared to SS rats, despite no differences in blood pressure between the two groups. These findings suggest a new role for PON-1 in regulating renal inflammation and fibrosis in the setting of chronic renal disease independent of blood pressure.

## 1. Introduction

Chronic kidney disease (CKD) affects >19 million people in the United States, is responsible for substantial cardiovascular long-term morbidity and mortality, and is predicted to double within the next 10 years [1,2,3,4]. CKD is a multifactorial progressive inflammatory disease that contributes to the increased cardiovascular burden. In addition to the classical risk factors for CKD, inflammation plays a central role in the onset and progression of renal injury in CKD [5,6,7,8]. Increased oxidant production and decreased antioxidant defense mechanisms are often detected in patients with CKD. Our lab and others have demonstrated that cardiotonic steroids (CTS) are significantly elevated in CKD and are important mediators of this inflammation and oxidative stress in this setting [9,10]. CTS enhances renal inflammation and fibrosis upon binding and signaling through the Na^+^/K^+^-ATPase [9,10,11], and chronically elevated levels of CTS also have deleterious effects on the progression of renal and cardiovascular disease [12,13,14].

Paraoxonase-1 (PON-1) is a hydrolytic lactonase enzyme that is synthesized in the liver and circulates in the bloodstream while attached to high-density lipoproteins (HDL) [15]. Clinical data have demonstrated an association between diminished lactonase activity of circulating PON-1 and adverse outcomes in the setting of CKD [15,16]. Evidence suggests a potential role for PON-1 in mediating cardiovascular disease (CVD) in patients with CKD [17]. Many studies have shown an association between diminished circulating PON-1 activity and adverse cardiovascular outcomes in the setting of CKD [15,18]. Lower PON1 is associated with low thiols, higher CRP, lower HDL3, and adverse future cardiovascular disease (CVD) outcomes [19,20]. Case–control studies showed that CKD patients have significantly lower PON-1 concentration compared to age and sex-matched controls [16]. These observations suggest that PON-1 could have a renal protective role that contributes to renal disease development and progression. Despite the clinical findings, there is very limited knowledge regarding any mechanistic basis for the role of PON-1 in the pathophysiology of CKD. Further, the precise means whereby decreased circulating PON-1 leads to adverse clinical events in CKD is not fully understood. Patients with CKD often developed progressive renal compromise, which leads to recurrent hospitalizations and poor clinical outcomes [21]. Renal damage represents one of the main independent risk factors for clinical deterioration and all-cause mortality [22]. As our understanding of the mechanistic links of CKD is lacking, there are restricted treatment options beyond the traditional treatment strategies. In this context, we sought to determine the role of diminished PON-1 as a potential factor in the development and progression of renal diseases in a well characterized model of high-salt induced renal disease.

## 2. Materials and Methods

### 2.1. Animals

All animal studies were performed in accordance with the National Institutes of Health’s Guide for Care were approved by the Institutional Animal Care and Use Committee at the University of Toledo (108692-02-UT). Control Dahl salt-sensitive rats (SS^Mcwi^, hereafter called SS rats) were used along with PON-1 mutant rats (designated SS-Pon1^em1Mcwi^, hereafter called SS-Pon1 KO rats) which were generated by injecting a CRISPR targeting the sequence AGTATTTTTCCAGGCTTACTGG into SS rat embryos. The resulting mutation is a 7 bp frameshift insertion in exon 4. Founder animals were genotyped by the Cel-1 assay and confirmed by sanger sequencing. The founders were then backcrossed to the parental strain, and subsequent litters were genotyped by fluorescent genotyping. There were 547 embryos injected, 302 transferred, 18 recipients (many had no pups), and 42 pups born (6 founders). All recipients were SD rats from Charles River. Age-matched, ten-week-old male rats were maintained on a high-salt diet (8% NaCl, Envigo, Teklad diets, Madison, WI, USA) for up to 5 weeks to initiate the salt-sensitive hypertensive renal disease that is characteristic of this model. The genotype of all animals enrolled in the study protocol was confirmed by DNA sequencing.

### 2.2. Biochemical Assays

Circulating PON lactonase activity was measured in rats’ sera, using a commercially available fluorometric assay (BioVision Incorporated, catalog # K999-100, Blvd, Milpitas, CA, USA) as previously described [15]. Briefly, PON lactonase activity was calculated as the hydrolytic activity toward a fluorogenic benzopyran-2- in the presence and absence of a specific PON inhibitor (2-hydroxyquinoline) as per the manufacturers’ protocol.

### 2.3. Western Blot Analysis

Proteins from renal tissue were homogenized in ice-cold radioimmunoprecipitation assay lysis buffer (pH 7.0; sc-24948; Santa Cruz Biotechnology, Santa Cruz, CA, USA) supplemented with freshly prepared Halt Protease and Phosphatase Inhibitor Cocktail (78446; Thermo Scientific, Waltham, MA, USA). Tissue homogenate was centrifuged at 15,000× *g* for 15 min at 4 °C. The supernatant was separated for protein quantification, and a total of 30 μg protein was used for detection. Proteins were resolved via SDS-PAGE under reducing conditions unless otherwise noted. After gel electrophoresis, the proteins were electrotransferred from the gel onto polyvinylidene difluoride (PVDF) membranes (0.45-μm PVDF Transfer Membrane; Thermo Scientific). Then, the membrane was blocked with Rapid Block TM solution (VWR Life Science, Radnor, PA, USA) and probed with the indicated antibody. Images were obtained using the Syngene Western Blot Imager G: BOX (Syngene, Cambridge, UK) and analyzed by ImageJ software (NIH, Bethesda, MD, USA).

### 2.4. Histology

The kidneys were fixed in 4% formaldehyde (pH 7.2) paraffin-embedded and cut into 4 μm sections. These tissue sections were deparaffinized with xylene and then rehydrated by sequential incubations in ethanol and water. 8-Oxo-2′-deoxyguanosine and CD68 antibodies were purchased from Abcam (Cambridge, MA, USA). A Vectastain Elite-ABC kit (Vector Labs) (Burlingame, CA, USA) was used following the manufacturer’s protocol. Trichrome and H&E staining for the kidney was then conducted on the 4 μm kidney tissue sections. For each 4 μm section, 10 images were randomly taken with a bright-field microscope with a 20× lens. Quantitative morphometric analysis was performed using automated and customized algorithms/scripts for batch analysis (ImageIQ Inc., Cleveland, OH, USA) written for Image Pro Plus 7.0, as we have described in detail. Renal histology was then graded in a blinded fashion by a pathologist (A.G.) and scored on a scale of 0 to 4 for glomerular hypercellularity, protein casts, and interstitial inflammation.

### 2.5. Reverse Transcription-Polymerase Chain Reaction (RT-PCR) and RNA Isolation

RNA extraction, cDNA preparation, and RT-PCR were all performed utilizing the QIAGEN (Germantown, MD, USA) automated workflow system which utilizes the QIAgility and QIAcube HT liquid handling robots. RNA from the kidney tissue was isolated utilizing the QIAzol/Chloroform extraction methodology via automated liquid handling equipment (Germantown, MD, USA). Approximately 500 ng of extracted RNA was used to synthesize cDNA (QIAGEN’s RT2 First Strand Kit cat #330404). RT-PCR was then performed utilizing QIAGEN’s Rotor-Gene Q thermocycler. The calculation of gene expression was conducted by comparing the relative change in cycle threshold value (ΔCt). Fold change in expression was also calculated using the 2-ΔΔCt equation as previously described [11]. The following rat Taqman primers were used and obtained from Thermo Fisher Scientific (Waltham, MA, USA): Timp-1 (Rn00587558_m1), TGFb (Rn00572010_m1), Col1A1 (Rn01463848_m1), IL-6 (Rn01410330_m1), and CCL2 (Rn00580555_m1). For the normalization of transcript expression, 18s rRNA obtained from Thermo Fisher Scientific was used as a housekeeping gene (catalog no. 4319413E).

### 2.6. 8-Oxo-2′-Deoxyguanosine Measurement in Urine

8-Oxo-2′-deoxyguanosine (8-OHdG) in 24-h urine samples was measured by ELISA purchased from Biovision (Milpitas, CA, USA) and performed in accordance with the manufacturers’ protocol.

### 2.7. Blood Pressure

Arterial blood pressure (BP) was measured using a volume-pressure recording tail-cuff method (CODA non-invasive BP system, Kent Scientific Corporation, Torrington, CT, USA) [23,24]. Conscious rats were allowed to acclimatize for 15–20 min. Animals’ temperatures were maintained between 30–35 °C before acquiring blood pressure readings. Ten acclimation cycles, followed by a minimum of 20 measurement cycles were recorded. Any movement or sign of stress during measurement were noted and measurement excluded. BP was also assessed by radio telemetry for 14 h following 4 weeks of 8% HS diet. Values of BP obtained by the two methods were highly correlated. Rats were euthanized after obtaining BP measurements; total body weights and organ weights were collected; and kidneys were collected and portions were either formalin-fixed or flash frozen for both histologic and molecular analysis.

### 2.8. Glomerular Filtration Rate

Estimation of glomerular filtration rate (eGFR) was measured as previously described [25]. Briefly, 7.5 mg/100 g body weight of FITC-sinistrin (Fresenius-Kabi, Graz, Austria) dissolved in sterile saline was injected via the tail vein. Transcutaneous FITC-sinistrin signal was measured for two hours via transdermal probes (Medibeacon, Mannheim, Germany) fixed on the back of the animals. The excretion kinetic curve was analyzed and GFR was calculated using the following equation:

(1) $$\mathrm{GFR}\;\left(\mathrm{mL}/\min/100\;g\;b.w.\right)=\frac{21.33\;\left(\mathrm{mL}/100\;g\;b.w.\right)}{t1/2\;\left(\mathrm{FITC}-\mathrm{sinistrin}\right)\left(\min\right)}$$

### 2.9. Statistical Analysis

Data are presented as the mean ± standard error of the mean. Student’s Unpaired *t*-test was used to assess statistically significant differences between the two groups. One-way ANOVA and post hoc multiple comparisons tests were used when comparing more than two groups. Kaplan–Meier estimates with the log-rank statistic were applied for mortality analysis. All statistical analyses were performed using GraphPad Prism 6 software. Statistical significance was accepted as *p* < 0.05.

## 3. Results

### 3.1. Targeted Editing of the PON-1 Locus Using CRISPR

**Figure 1:**
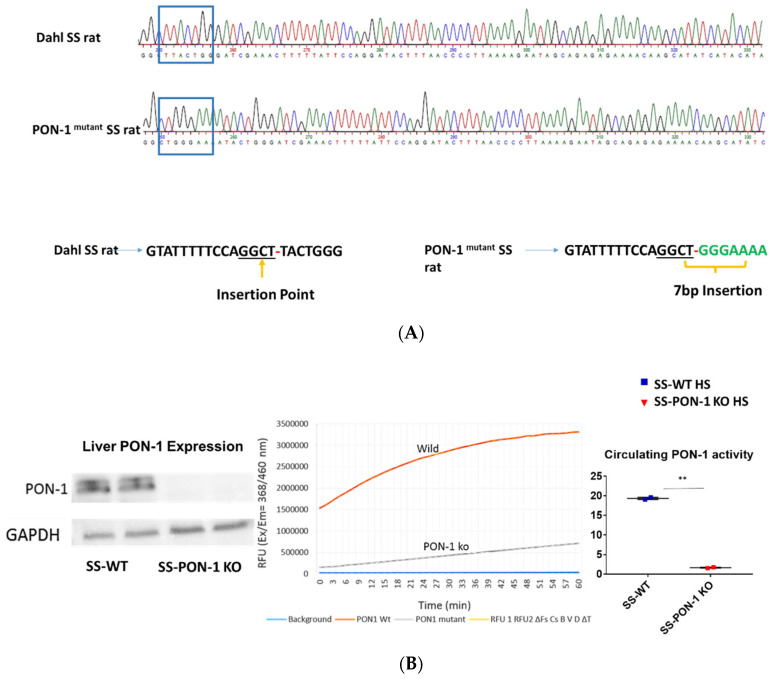
Screening animals for targeted mutation at the PON-1 locus. (**A**) Tail DNA samples were screened by Cel-1 assay and confirmed by Sanger sequencing. (**B**) Immunoblot analysis of PON-1 protein in liver from SS-PON-1 KO mutant and SS rats (N = 2) and Circulating PON-1 activity assay analysis in plasma collected from SS-PON-1 KO rats and SS rats (N = 2). Liver expression of PON-1 is abolished in SS-PON-1 KO rats compared to SS-wild rats PON-1 and leads to significantly decreased circulating PON-1 activity. ** *p* < 0.01 vs. high-salt.

To investigate the role of PON-1 in hypertensive renal disease using a mutant rat model of PON-1 CRISPR was designed to target the sequence GGCTTACTGGG in SS rat embryos. Injection of CRISPR into single-cell S embryos resulted in the generation of a founder rat with 7 bp frameshift insertion in exon 4. Founder animals were genotyped by the Cel-1 assay and confirmed by Sanger sequencing (Figure 1A). The founders were then backcrossed to the parental strain and subsequent litters were genotyped by fluorescent genotyping. There were 547 embryos injected, 302 transferred, 18 recipients (many had no pups), and 42 pups born (6 founders). All recipients were SD rats from Charles River. Western blot analysis and serum activity assay confirmed the mutant status of PON-1. PON-1 protein was not detectable in the liver tissue collected from the SS-PON-1 KO rats and circulating serum PON lactonase activity was significantly diminished compared to SS rats (Figure 1B).

### 3.2. Targeted Disruption of PON-1 Affects Phenotype and Survival Outcome in CKD Model

**Figure 2:**
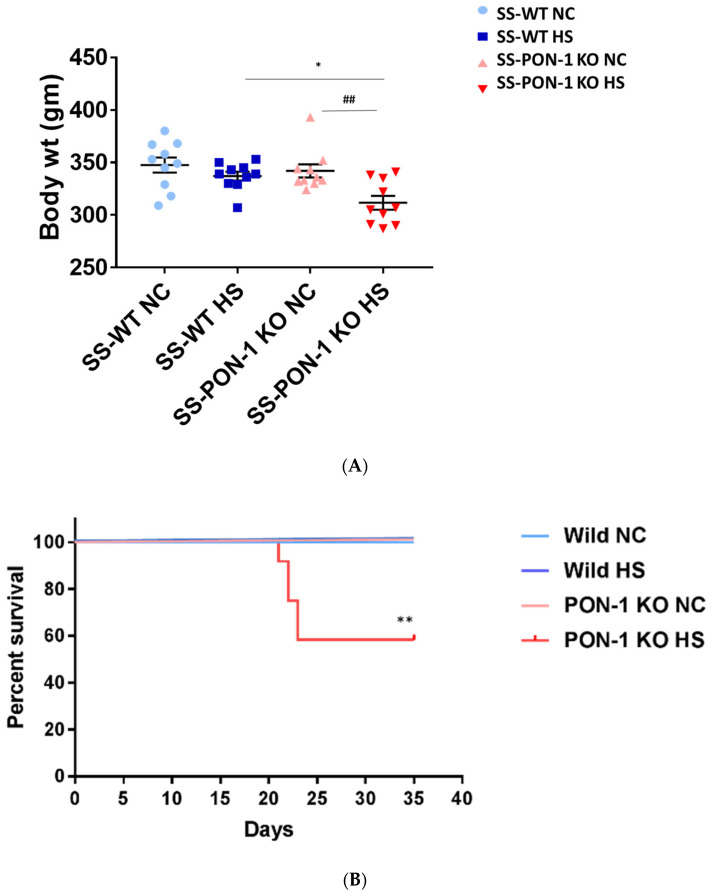
Targeted mutation of Paraoxonase-1 leads to phenotype changes and increased mortality. (**A**) phenotypic characteristics; SS-PON-1 KO showed significant decrease in body weight compared to SS rats after 5 weeks on high-salt diet (N = 10). (**B**) Early mortality observed in SS-PON-1 KO rat models after average of 33 days of high-salt diet (8% NaCl), while no mortality was observed in SS rats (N = 12). * *p* < 0.05, ** *p* < 0.01 vs. high-salt, ^##^ *p* < 0.01 vs. normal chow.

At the start of the study, both SS rats and SS-PON-1 KO rats demonstrated a comparable body weight. After 5 weeks of high-salt diet, and upon inspecting for phenotypic changes, we observed a difference in body weight. While there was a significant reduction in the body weight in SS-PON-1 KO compared to SS rats on high-salt diet, no changes in body weight were detected in SS-PON-1 KO compared to SS rats on low-salt diet (Figure 2A). Further, SS-PON-1 KO rats on high-salt diet demonstrated significant mortality (mean length of time until death = 33 days), whereas no mortality was observed in SS rats on high-salt diet, as well as SS-PON-1 KO and SS rats on low-salt diet (Figure 2B).

### 3.3. Targeted Disruption of PON-1 Significantly Increases Renal Fibrosis in Hypertensive Renal Disease

**Figure 3:**
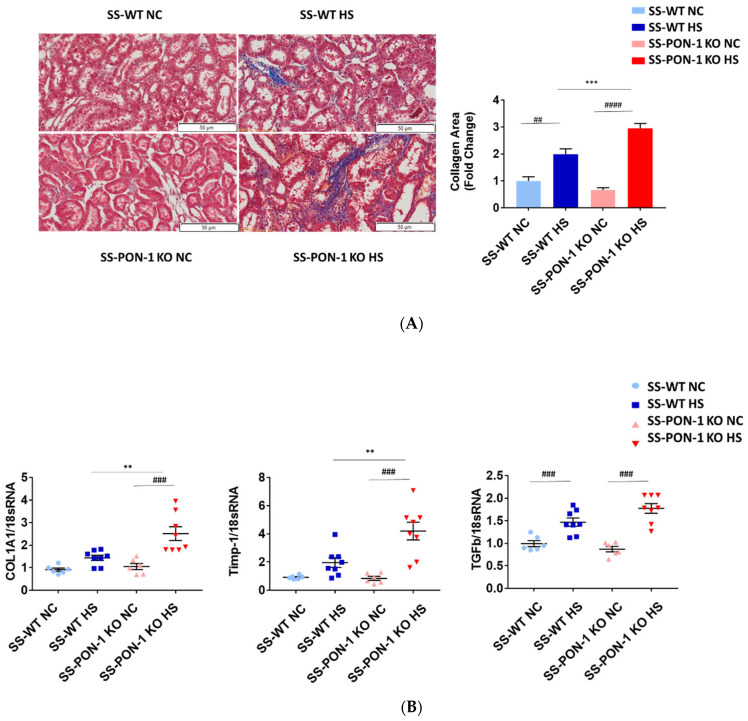
Targeted mutation of paraoxonases-1 contributes to increase fibrosis in renal tissue as measured by (**A**) collagen area (Pixels) (N = 6) and (**B**) key fibrotic gene expression in renal tissue. Quantitative PCR from Dahl SS-PON-1 KO and SS rats after 5 weeks high-salt diet (8% NaCl) or normal chow (HS N = 8, NC N = 6). SS-PON-1 KO rats show a significant increase in renal fibrosis, compared to SS rats following 5 weeks 8% high-salt diet, ** *p* < 0.01, *** *p* < 0.001 vs. high-salt, ^##^ *p* < 0.01, ^###^ *p* < 0.001, ^####^ *p* < 0.0001 vs. normal chow.

Kidneys of rats were sectioned and trichrome stained to examine them histologically for evidence of renal fibrosis. Here, we noted that SS-PON-1 KO rats had more renal fibrosis at 5 weeks compared to the WT controls (Figure 3A). Next, we performed a quantitative real-time PCR array on kidneys from both SS-PON-1 KO and SS rats. Here, we noted that kidneys from SS-PON-1 KO demonstrate a significant increase in the expression of key genes related to fibrosis (COL1A1, TGFb1, and Timp-1) compared to kidneys collected from SS rats (Figure 3B).

### 3.4. Targeted Mutation of PON-1 Significantly Increased Renal Inflammation in Hypertensive Renal Disease

**Figure 4:**
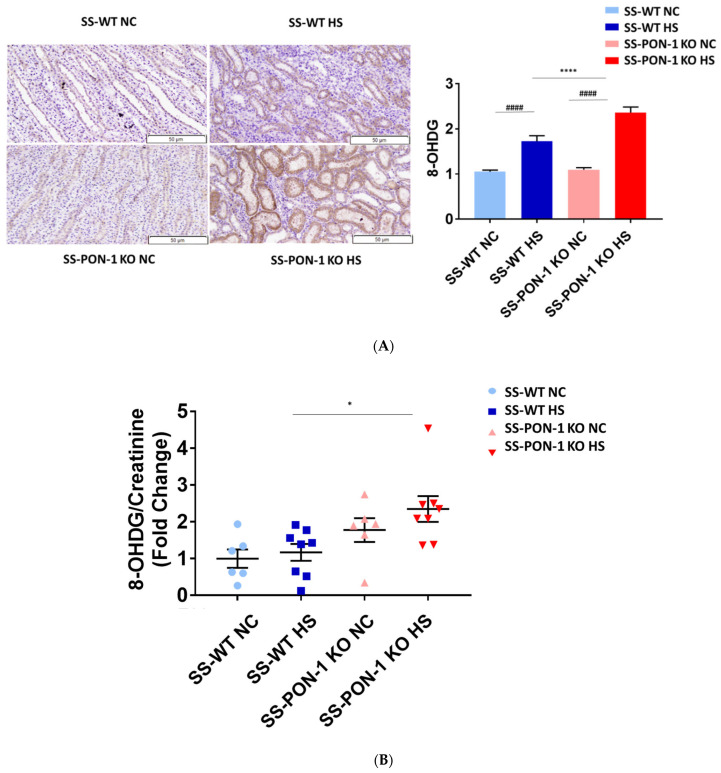

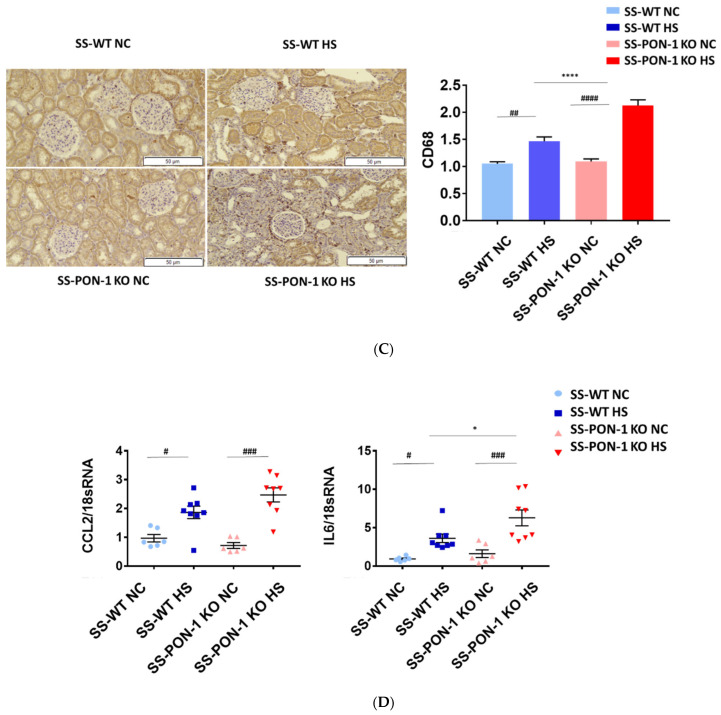
Targeted mutation of paraoxonases-1 contributes to an increase in renal oxidative stress as demonstrated by (**A**) 8-Oxo-2′-deoxyguanosine (8-OHdG) histology, (**B**) urinary excretion of 8-OHdG, (**C**) recruitment of CD68 positive immune cell in renal interstitium, and (**D**) increased expression of key inflammatory gene in renal cortical tissue. SS-PON-1 KO rats show significant increase in renal inflammatory markers, compared to SS rats following 5 weeks 8% high-salt diet (HS N = 8, NC N = 6). * *p* < 0.05, **** *p* < 0.0001 vs. high-salt, ^#^ *p* < 0.05, ^##^ *p* < 0.01, ^###^ *p* < 0.001, ^####^ *p* < 0.0001 vs. normal chow.

To examine the role of PON-1 in mediating oxidative stress, kidneys of rats were sectioned at 5 weeks and stained for 8-Oxo-2′-deoxyguanosine (8-OHdG) to examine evidence of renal oxidative stress. SS-PON-1 KO rats demonstrated significantly higher levels of oxidative stress in the kidney compared to SS rats (Figure 4A). Likewise, twenty-four-hour urine collected from SS-PON-1 KO rats showed significantly higher excretion levels of 8-OHdG compared to urine collected from SS rats (Figure 4B). Next, to assess the role of PON-1 in mediating inflammation, we examined the recruitment of CD68 positive immune cells (i.e., macrophages) within the kidney. Kidneys from SS-PON-1 KO rats showed less macrophage infiltration compared to kidneys from the SS rats (Figure 4C). To further assess renal inflammation in this model, we performed a quantitative real-time PCR array to examine the expression of key inflammatory genes in kidneys from both SS-PON-1 KO and SS rats. Consistent with the histology, kidneys from SS-PON-1 KO demonstrate a significant increase in the expression of key genes related to inflammation (i.e., CCL2, IL6) compared to kidneys collected from SS rats on a high-salt diet (Figure 4D).

### 3.5. Paraoxonase-1 Have Significant Impact on Renal Function in Hypertensive Renal Disease

**Figure 5:**
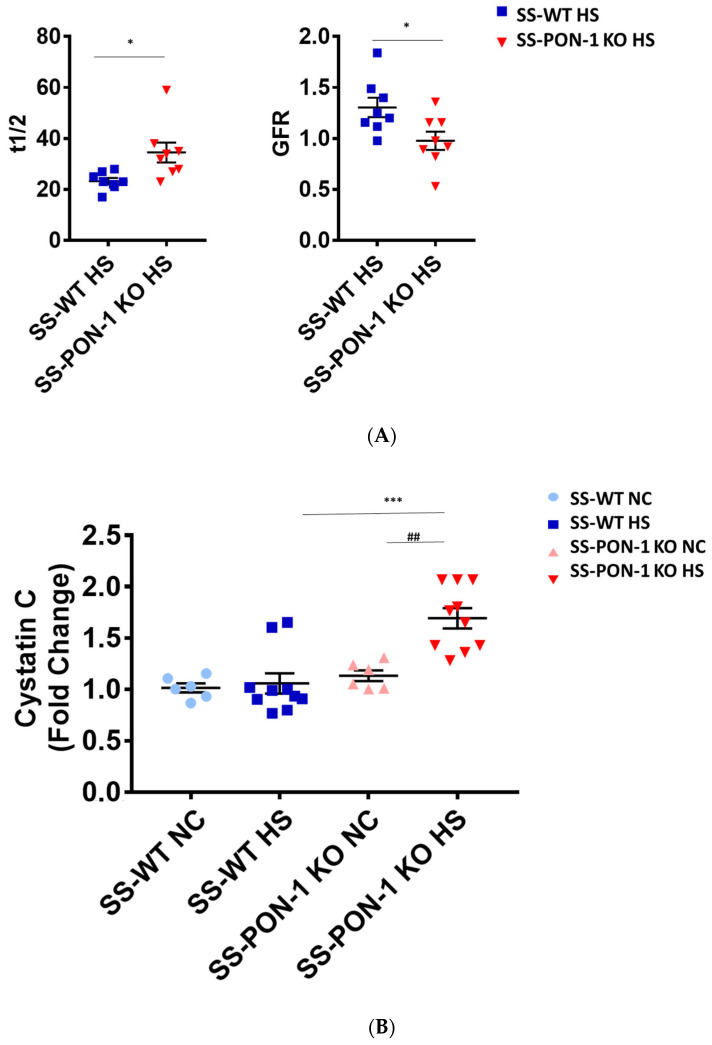

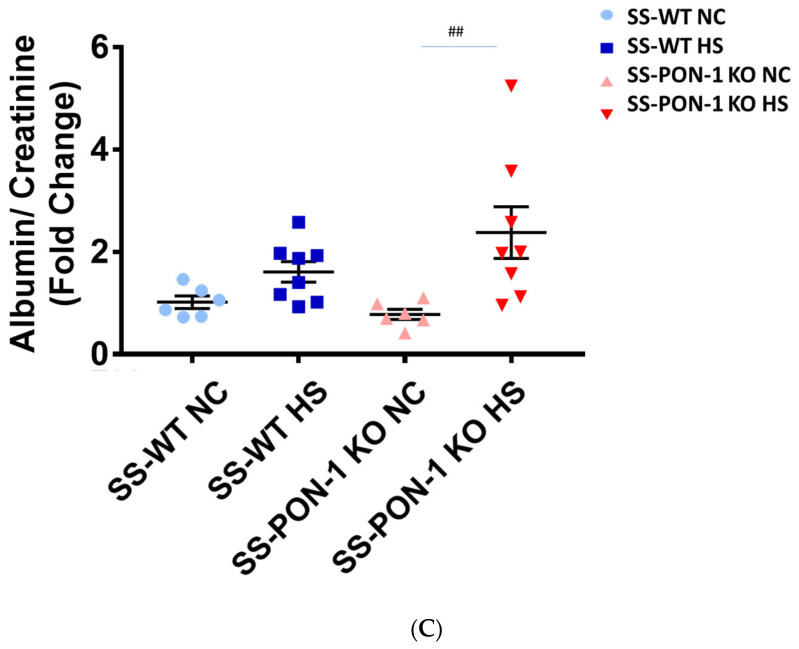
Targeted mutation of paraoxonases-1 contributes to a decline in renal function as assessed by (**A**) FITC-Sinistrin glomerular filtration rate, (**B**) plasma cystatin C measurement, and (**C**) albumin urinary excretion. SS-PON-1 KO rats show a significant decline in renal function compared to SS rats following 5 weeks 8% high-salt diet (HS N = 8, NC N = 6). * *p* < 0.05, *** *p* < 0.001 vs. high-salt, ^##^ *p* < 0.01 vs. normal chow.

We assessed renal function using FITC-Sinistrin transdermal probe to calculate glomerular filtration rate (GFR) in conscious rats. After 5 weeks on high-salt diet, SS-PON-1 KO rats demonstrated significantly decreased renal function compared to the SS rats as assessed by prolonged FITC-Sinistrin excretion mean half-life (t1/2) and decreased GFR (Figure 5A). We further examined renal function by measuring cystatin C in plasma, where SS-PON-1 KO rats had lower plasma cystatin C levels at 5 weeks compared to the WT controls (Figure 5B). In order to determine whether these changes in renal function were associated with renal injury, we measured urine albumin-to-reatinine ratio as a measure of albuminuria after 5 week on the high-salt diet. While SS rats showed no difference in albuminuria, SS-PON-1 KO rats showed a significant increase in albuminuria after the 5-week high-salt diet (Figure 5C).

### 3.6. Paraoxonase-1 Role as Renal Protective Factor in Hypertensive Renal Disease without Altering Blood Pressure

**Figure 6:**
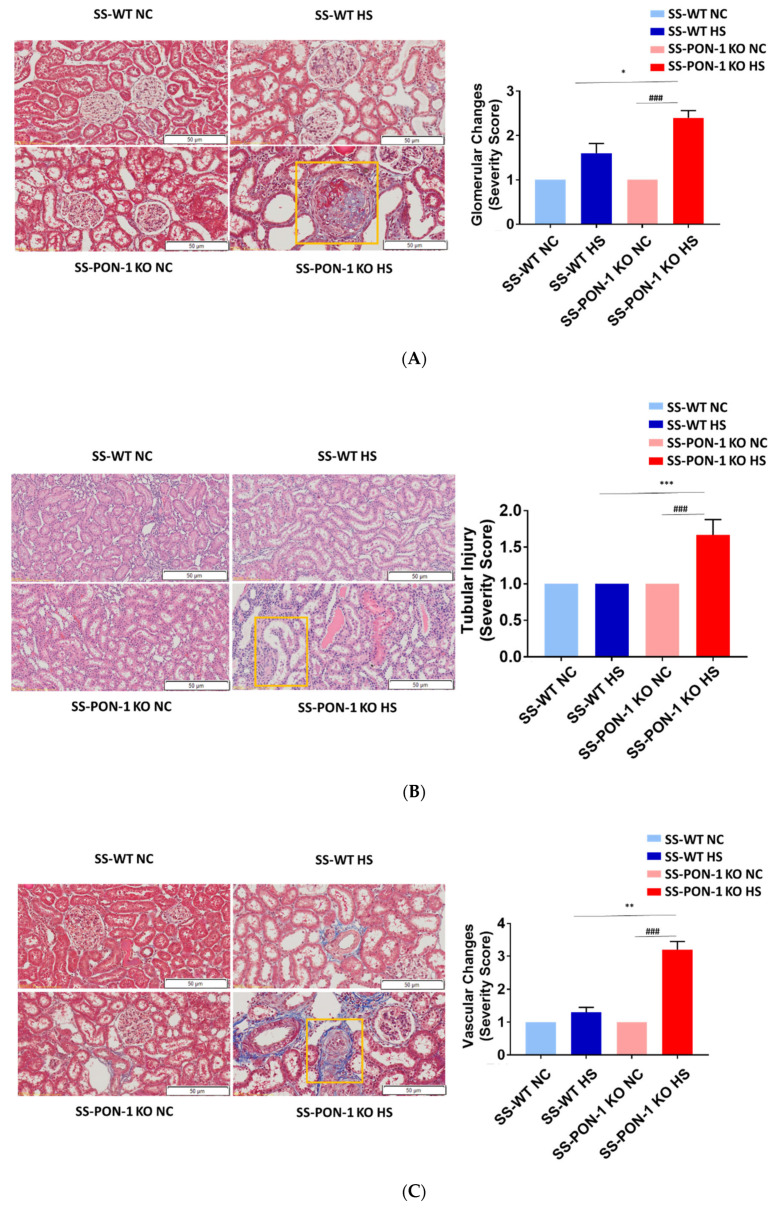
Targeted mutation of paraoxonases-1 leads to a significant increase in histological changes accompanying renal damage; (**A**) glomerular sclerosis, (**B**) tubular necrosis, and (**C**) vascular changes. Representative H&E histology (right) and quantification (left). SS-PON-1 KO rats show a significant increase in renal injury, compared to SS rats, following 5 weeks on the 8% high-salt diet (N = 6). * *p* < 0.05, ** *p* < 0.01, *** *p* < 0.001 vs. high-salt, ^###^ *p* < 0.001 vs. normal chow.

**Figure 7:**
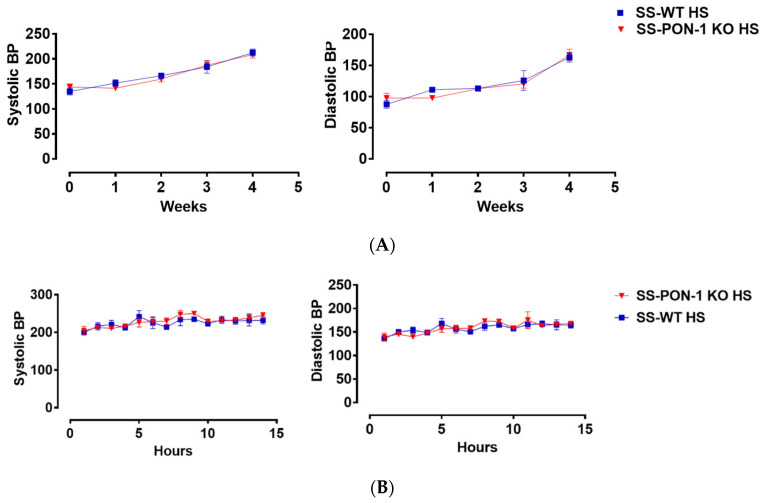
Targeted mutation of paraoxonases-1 shows no effect on blood pressure in the SS_PON-1 KO compared to SS rats after a high-salt diet. (**A**) Systolic (left) and diastolic (right) blood pressure assessed by tail cuff plethysmography (N = 8/group) over 4 weeks (**B**) Systolic (left) and diastolic (right) blood pressure assessed by radiotelemetry at 4 weeks (N = 4/group). SS-PON-1 KO rats show similar increase in blood pressure, compared to SS rats following 5 weeks 8% high-salt diet.

Next, we histologically examined kidneys for evidence of renal injury. After 5 weeks on the high-salt diet, histological examination demonstrated that SS-PON-1 KO rats had significantly higher levels of renal damage, represented by the presence of more glomerular sclerosis, acute tubular necrosis changes, and vascular changes, compared to kidneys from the SS rats (Figure 6A–C). Finally, to examine the effect blood pressure in the observed renal injury phenotype of SS-PON-1 KO rats, we used both tail-cuff plethysmography and radiotelemetry to assess potential differences in blood pressure between the two groups. At 4 weeks, both SS-PON1 KO and SS rats developed similar degrees of hypertension as assessed by both tail-cuff (Figure 7A) and radiotelemetry measured (Figure 7B) systolic and diastolic blood pressure.

## 4. Discussion

Chronic kidney disease (CKD) is a multifactorial disease with a complex etiology. Major risk factors such as inflammation have been demonstrated to be important in mediating renal disease progression. Even with advanced management strategies, patients with CKD often develop a chronic inflammatory state. Several observations suggest to us that PON-1 may play a central role in mediating the chronic inflammatory state underlying CKD. First, reduced activity of PON-1 is linked with increased oxidant stress in atherosclerosis settings, a shared feature underlying the pathogenesis of CKD [26,27]. Second, circulating PON-1 activity was found to be significantly lower in patients with CKD compared to healthy controls [9,15]. Third, diminished circulating PON-1 activity predicts future morbidity and mortality in patients with CKD [9,15]. Together, these findings suggest that PON-1 is renally protective and regulated during oxidative stress. Hence, in the current study, we evaluated PON-1 as a potential candidate molecule in CKD settings.

PON-1 is a hydrolytic lactonase enzyme that is synthesized in the liver and secreted into the serum as an HDL-associated protein. There is very limited knowledge regarding any mechanistic basis for the role of PON-1 in the pathophysiology of CKD. In the current study, we used a novel SS-PON-1 KO rat model to examine the role of PON-1 in a well-characterized model of high-salt induced renal disease. Control Dahl salt-sensitive rats (SS-wild) along with Pon1 mutant rats (SS-PON1 KO rats) were maintained on a high-salt diet (8% NaCl) to initiate the salt-sensitive hypertensive renal disease that is characteristic of this model. While this diet is generally well-tolerated in SS rats over 8–12 weeks [28,29], we demonstrated early mortality in SS-PON-1 KO rats (mean length of time until death = 33 days), while no mortality was observed in SS rats. Due to this unexpected mortality event, we limited the study period to 5 weeks to closely examine the impact of PON-1 on CKD progression. Indeed, SS-PON-1 KO rats had significant decreases in GFR as well as increases in plasma cystatin C, and albuminuria compared to SS rats after the high-salt diet. Upon examining the kidneys, SS-PON-1 KO demonstrated increased recruitment of CD68 positive immune cells in the renal interstitial, as well as increased expression of the key inflammatory genes (Timp-1, MCP-1, IL-6, COL1A1, and TGF-β) compared to the SS rats.

These findings highlighted the clinical importance of PON-1 in this high-risk population. Our findings are in agreement with previous studies reporting that that PON-1 directly suppresses macrophage pro-inflammatory responses and decreases sustained pro-inflammatory reactions [30]. Oxidative stress and inflammation play an interactive role in renal disease and are mutually associated with altered PON-1 activity in plasma of patients with renal disease [31]. Changed PON1 activities on different HDL subclasses and diminished anti-oxidative protection have been reported as important factors in the development of adverse clinical outcomes in CKD and ESRD patients [32]. Furthermore, SS-PON-1 KO demonstrated evidence of increased renal injury, noted by increased renal fibrosis, sclerosis, and acute tubular injury changes compared to SS rats. These findings support clinical data that implied a protective role of PON-1 in the setting of CKD [15,18,30]. A previous clinical study by Marsillach et al. suggested that a significant increase in serum PON-1 activity can improve oxidative stress in pre-dialysis patients with CKD [33]. Interestingly, SS-PON-1 KO rats also showed an increased renal oxidative stress, as demonstrated by both increased amounts of the oxidative stress marker 8-Oxo-2′-deoxyguanosine (8-OHdG) in the kidney and higher urinary excretion levels of 8-OHdG compared to SS rats. Importantly, the renal phenotype displayed could not be explained by changes in blood pressure, as there were no differences in blood pressure between SS-PON-1 KO and SS rats as measured by both tail-cuff plethysmography and radiotelemetry.

While for many years PON-1 has been implicated as a risk factor in atherosclerosis (reviewed [34]), we provide experimental evidence that PON-1 is a major risk factor in CKD progression through regulating renal inflammation and fibrosis in a well characterized model of high-salt induced renal disease. Augmenting endogenous counter-regulatory mechanisms such as PON-1 may provide a unique therapeutic opportunity to attenuate renal disease progression. These findings have expanded our knowledge regarding the physiological role of PON-1 as a renal protective enzyme. Modulation of PON-1 levels and activity may present a novel therapeutic break to modulate the inflammatory events which contribute to the initiation and progression of renal disease. Future studies need to investigate (1) the mechanisms through which PON-1 exerts its protective effects and (2) intervention studies to increase PON-1 activity and test whether it can be targeted therapeutically in patients with CKD.

## Data Availability

Data is contained within the article.
